# Measuring the Quality of Life and Psychological Distress of Dormitory Students at a University in Sharjah

**DOI:** 10.7759/cureus.73666

**Published:** 2024-11-14

**Authors:** Ahmed M Youssef, Muhamed Shamsaldin, Bushra Abuzayed, Shahad Alameeri, Mithaq M Al Eid, Ayah Dib, Amal Hussein, Amna Khalid

**Affiliations:** 1 College of Medicine, University of Sharjah, Sharjah, ARE; 2 Family and Community Medicine, University of Sharjah, Sharjah, ARE

**Keywords:** dormitories, psychological distress, psychology, quality of life, school psychology, university students

## Abstract

Objective: Living in university dormitories can have a negative impact on students' psychological health and quality of life. This cross-sectional study aimed to investigate the frequency and risk factors of psychological distress and quality of life among university students living in dormitories at a university in Sharjah.

Methods: A total of 336 participants between the ages of 18 and 24 years were recruited. The World Health Organization Quality of Life Brief Version (WHOQOL-BREF) scale and Kessler's Psychological Distress Scale (K10) were used to assess the quality of life (QoL) and psychological distress (PD), respectively. Data were analyzed via SPSS 25 (Chicago, IL: IBM Corp.) and p≤5% were reported as statistically significant.

Results: A total of 43.45% (n=145) of participants lived in dormitories, while 56.55% (n=186) lived elsewhere. The mean QoL was higher (94.55) for non-dormitory students compared to dormitory students (88.97) (p<0.000, 95% CI = -8.591 to -2.577). Our results also show that the QoL is inversely proportional to PD (p<0.001).

Conclusion: The findings of this study indicate that students living in dormitories are at higher risk of experiencing lower QoL compared to those living elsewhere, on top of the high rates of PD that they have to endure.

## Introduction

Psychological distress (PD) is defined as a condition where an individual experiences unpleasant feelings and appraises his circumstances as taxing or exceeding his or her resources [1]. Quality of life (QoL) is defined as “the way an individual views their standing in society, considering the cultural norms, values, objectives, expectations, standards, and concerns that shape their life” [2].

University students are shifting from high school to university which can be a difficult adjustment. This is a transitional stage to adulthood that is characterized by dramatic changes in behavior, thought, and perceptions about the world [3]. These students will be required to deal with a plethora of difficulties including a new lifestyle that requires quick adaptation, a strict timetable, and a combination of academic responsibilities that does not forgive the slow among them [4]. All of this change is anticipated along with an expectation from them to act more maturely as they have graduated from high school [5]. The effects of these challenges are well illustrated in the higher rates of mental illnesses found among university students. The literature shows that as much as 33% of university students are depressed [6]. Although the challenges of this transitional period are shared among university students. However, students living in university dormitories are likely to face extra tension due to several factors. Some of these factors are living alone at a distance from the usual social support networks, necessitating greater self-reliance, contending with various stressors [7], adapting to a new lifestyle, coping with irregular sleeping and waking hours, grappling with uncertainties about their future job prospects, navigating educational obstacles, struggling with a lack of interest in discipline, and dealing with a high volume of classes and lesson [8]. All these tension factors collectively contribute to the complex web of challenges that university students, particularly those living in dormitories, confront during this transitional phase, ultimately impacting their overall quality of life.

The impact of this is well documented in previous literature. Many studies suggest that those living in dormitories are less satisfied with their lives with one study held in Beijing stating that the students’ overall dissatisfaction with the dormitory environment was high (81%), especially in terms of air quality and acoustic environment [9]. A study found that living in a dormitory and separation from family increases stress severely (60%), potentially leading to suicidal thoughts among students [10]. The rates of depression are higher in students living in dormitories with a recent study discovering that as much as (30%) of students living in the hostel were suffering from varying levels of depression [11].

To the best of our knowledge, this is the first study to provide valuable information on the anxiety levels among dormitory university students across the UAE comparing it with day scholars. This study aimed to compare the levels of psychological distress between university students residing at home and in dormitories. It also explores the effect of living in dormitories on psychological distress and quality of life among university students in Sharjah.

## Materials and methods

Settings and population

This study was conducted at the medical campus of a university located in the United Arab Emirates. The study’s target population was undergraduate students enrolled at medical, dentistry, or pharmacy colleges. Students aged between 18 and 24 years enrolled in study years one, two, or three of their respective college curricula were recruited. As for dormitory students, those who lived within the emirates of Dubai, Sharjah, and Ajman were included in the study. Students who lived outside the aforementioned emirates or had lived in the dormitories for less than one year were also excluded from the study. In regards to non-dormitories students, students were excluded from the study if they were older than the age of 24 years. The data were collected between January and March 2020. The data collected were not affected by the COVID-19 pandemic-related subsequent lockdown since all the collection was completed before the lockdown in the UAE.

Sample size calculation

The minimum sample size needed to conduct this study was calculated using Cochran’s formula as follows: n=z^2^ p (1-p)/d^2^, where n is the minimum sample size needed, z is the z score for the desired confidence level, p is the expected prevalence, and d is the level of precision or margin of error. For a confidence level of (95%) and a precision level of (5%), the minimum sample size needed was 248 students. To ensure adequate comparative analysis, it was decided that 124 students should be living in the dormitories, and the remaining 124 students ought to be living outside of the dormitories.

Study design

In this cross-sectional comparative study, we opted for a non-probability purposive sampling method due to its practicality in accessing our target population, i.e., students within the medical campus of the university and the dormitory complexes. Printed copies of the questionnaires along with the information sheet and consent form were distributed to the participants. The self-administered multiple-choice questionnaire ensured the privacy of the respondents, fostering candid responses. The questionnaires were in English language and there was no need to translate them.

The questionnaire was developed using the World Health Organization Quality of Life Brief Version (WHOQOL-BREF) scale which has 26 questions about physical and psychological health, social relations, and the environment of the individual [12]. Kessler’s Psychological Distress Scale (K10) was used with 10 questions on emotional stress scored on a five-point Likert scale [13]. Kessler’s psychological distress scale (K10) categorizes the participants into four groups according to their likelihood to be psychologically healthy, and to have a mild, moderate, or severe psychological disorder. Moreover, we have added a few extra questions relating to demographic data like age and gender, field of study, year of study, and whether or not they are living in dormitories. We also added additional questions for students to rate their satisfaction with the dormitories and their quality of life.

Data analysis

Data were analyzed using IBM SPSS Statistics version 25.0 (Chicago, IL: IBM Corp.), for efficient entry, cleaning, and comprehensive analysis of the acquired information. Statistical analysis was conducted to address both the primary and secondary objectives of this study.

Primary Objective Analysis

To compare the quality of life (QoL) and psychological distress between dormitory students and non-dormitory students, independent samples t-tests were used for normally distributed continuous variables. For non-normally distributed continuous variables, the non-parametric Kruskal-Wallis test was applied. The chi-square test was used to assess associations between categorical variables, such as living conditions and specific demographics.

Secondary Objective Analysis

Pearson correlation was used to explore the relationship between continuous variables, such as the relationship between QoL scores and psychological distress levels. Additionally, ANOVA was employed to compare the mean differences in QoL and psychological distress across multiple demographic subgroups (e.g., age groups or year of study). A p≤0.05 was considered statistically significant for all analyses.

Ethical consideration

Prior to participation, each student provided informed consent, with the freedom to withdraw from the study at any point during data collection. In the event of withdrawal, the participant's data were promptly discarded. No identifying information was gathered from the participants. Ethical approval was secured from the Medical Research Ethics Committee of the College of Medicine and Health Sciences.

## Results

Demographics

Of the 340 responses, 336 were included with the exclusion of four participants due to incomplete questionnaires. Table 1 describes the demographic characteristics of the participants. A total of 250 (74.4%) participants were females resulting in a female-to-male ratio of 3:1. The mean age of the sample was 19.62±1.03. Of the total participants, 138 were from the College of Dentistry, 109 were from the College of Medicine, and 89 were from the College of Pharmacy. A little more than half of the participants (56.55%) were not living in the university dormitories as shown in Table 1.

**Table 1:** Demographics of participants.

Demographics	Frequency (n)	Percentage
Age (years) (mean±SD=19.62±1.03)	-	-
Gender		
Male	86	25.6%
Female	250	74.4%
Field of study		
Medicine	109	32.44%
Dentistry	138	41.07%
Pharmacy	89	26.49%
Academic year		
Year 1	109	32.44%
Year 2	139	41.67%
Year 3	88	25.89%
Are you living in the university dormitory?		
Yes	146	43.45%
No	190	56.55%
Students’ satisfaction with living in the dormitory		
Very unsatisfied	47	14%
Unsatisfied	54	16%
Neither unsatisfied nor dissatisfied	112	33.5%
Satisfied	106	31.5%
Very satisfied	17	5%

Quality of life

Before assessing the quality of life of the participants using a standardized questionnaire, we asked the participants to roughly rate their quality of life. The majority of the participants (55.7%) reported having a good quality of life, while 23.5% reported having a very good quality of life. Only a small percentage (4.2%) perceived having a poor or very poor quality of life.

**Figure 1:**
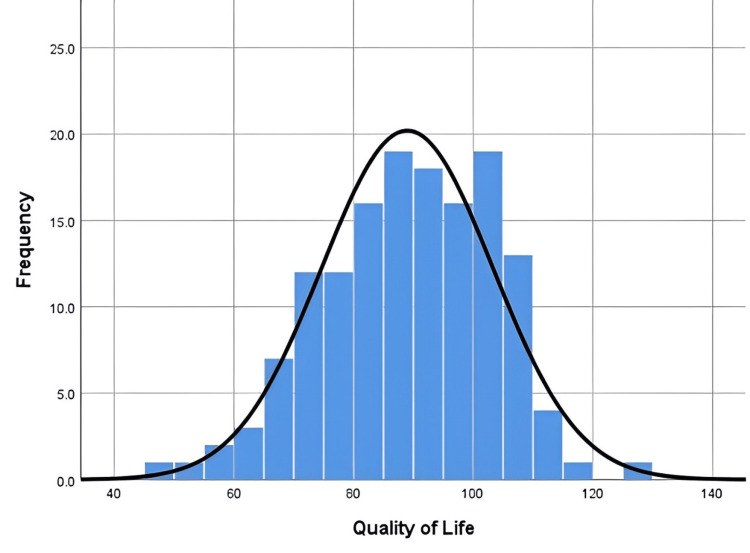
Quality of life histogram distribution for students living in the dormitories. Mean: 88.97; standard deviation: 14.314; N: 145

**Figure 2:**
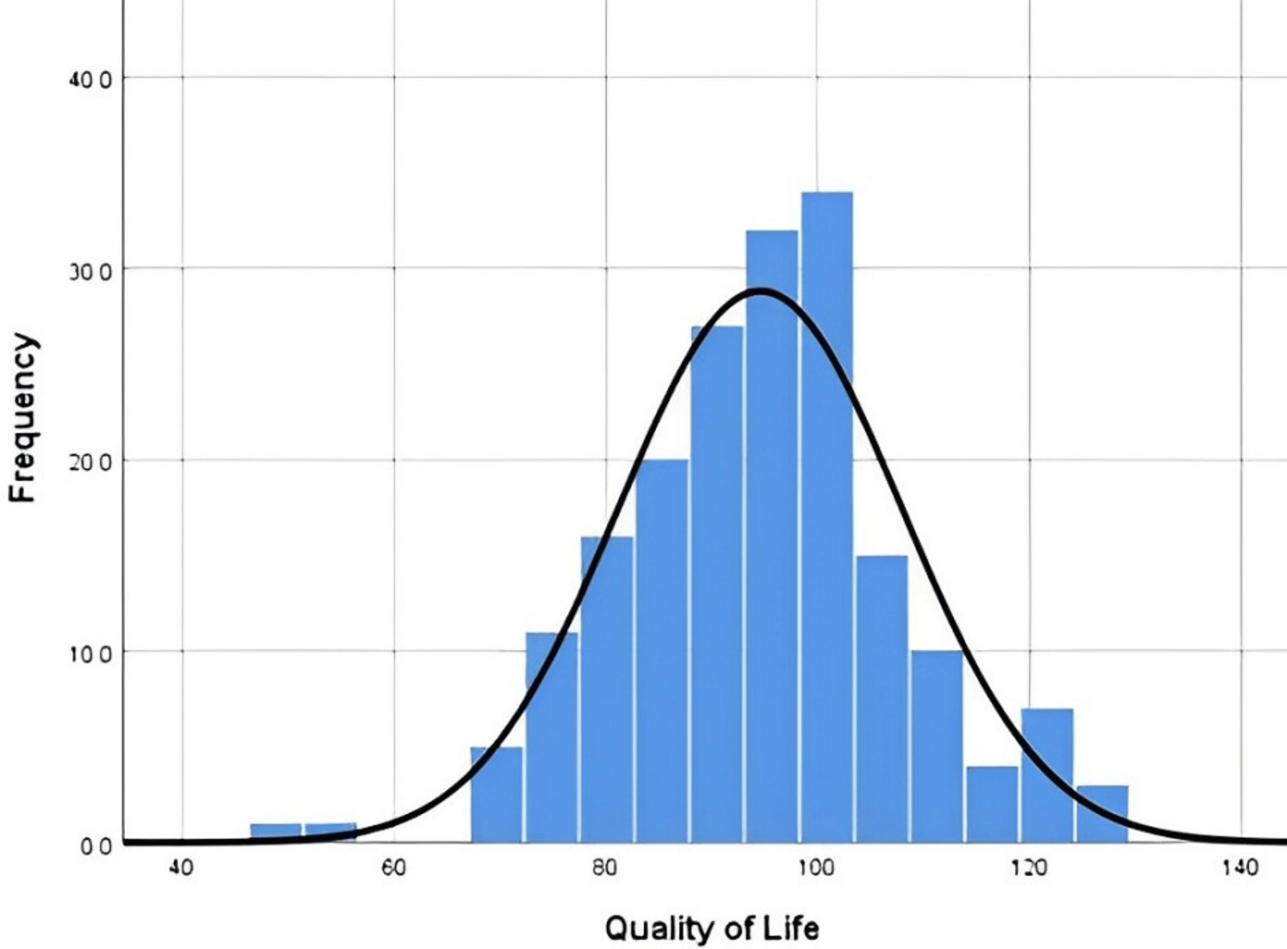
Quality of life histogram distribution for students living outside of the dormitories. Mean: 94.55; standard deviation: 13.383; N: 186

Scores obtained by the participants in different domains of QoL are illustrated in Figures 1, 2. Significant differences were observed in all QoL domains between mean QoL among non-dormitory students and dormitory students (p<0.001, 95% CI = -8.591 to -2.577) with non-dormitory students reporting higher mean (94.55) compared to dormitory students (88.97). Quality of life scores did differ significantly between male and female students with females reporting a poorer quality of life (90.68) than males (96.22) (p<0.002, 95% CI = 2.112, 8.975).

There is a correlation between having a roommate and the quality of life of the participants. The quality of life mean is higher for those who had no roommate (92.92) compared to those living with a roommate (87.13) (p<0.023). No significant differences between the quality of life and the city where the participants’ families live were observed (p<0.2815).

Psychological distress

A total of 32.7% of the study participants were likely to have a severe psychological disorder. Furthermore, 22.6% were predicted to have a moderate disorder, 22.3% were shown to have a mild disorder, and lastly, 22.3% of the participants were predicted to be well without a psychological disorder.

The comparison of the prevalence of severe psychological distress between the two genders revealed that a higher percentage of females (35.6%) were present in the severely disordered group as compared to males (24.4%) using the chi-square test (χ^2^=13.571, df=3, p=0.004). However, the difference in rates of severe psychological disorder was not statistically different in dormitory and non-dormitory students. 

Looking at the majors of the participants, those from the College of Pharmacy had the highest percentage of potentially severe disorders (43.8%). This is followed by the College of Medicine, of which 33.9% had potentially severe disorders. Lastly, the College of Dentistry had 24.6% of the severely disordered sample.

It was found that 19.2% of those who visited their families at least once a week were categorized as having a severe disorder while an astounding 68.8% of those who visit their families once every four months are likely to have a severe psychological disorder (χ^2^=29.860, df=18, p=0.039).

Our study found that individuals who never exercise showed a higher likelihood (40.1%) of belonging to the severe disorder group compared to those engaging in physical activity at least five times a week (25.0%). However, there was no significant correlation between not exercising and being in a severely psychologically distressed group (χ^2^=14.638, df=9, p=0.10).

**Figure 3:**
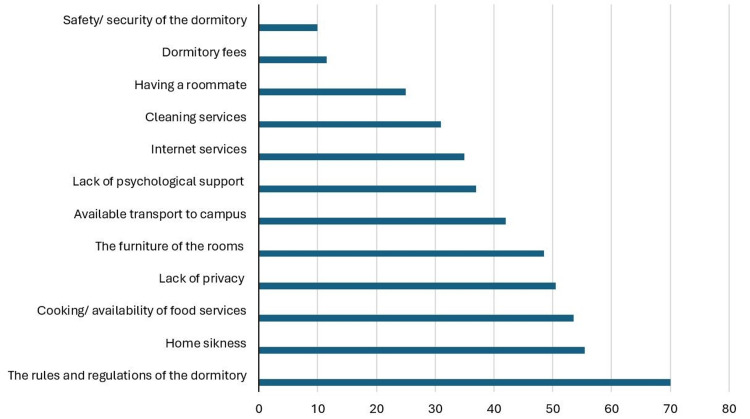
Aspects related to the dormitories that students believe contribute negatively to their lives. X axis: number of students who believe this factor contributed negatively to their lives. Y axis: factors related to dormitories that students believe contributed negatively to their lives.

Things that contribute negatively to participants’ lives living in the dormitory are illustrated in Figure 3. The most chosen option was the rules and regulations of the dormitory (70%). This was followed by homesickness (55.4%) and the availability of food services (53.6%). On the other hand, the factors that had a minor negative effect included the safety of the dormitories (10%), and dormitory fees (11.5%).

Quality of life and psychological distress

**Figure 4:**
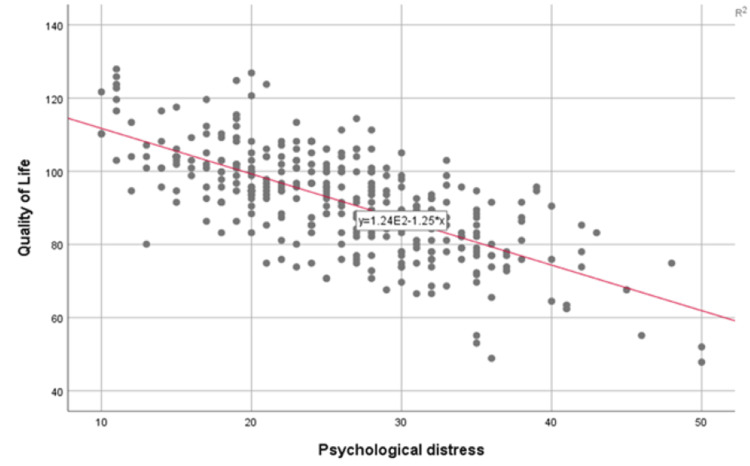
Correlation between psychological distress and quality of life among dormitory students. X axis: score of Kessler’s Psychological Distress Scale (K10) (range: 10-50); Y axis: score of the World Health Organization Quality of Life Brief Version (WHOQOL-BREF) scale (range: 40-140).

The QoL was found to be inversely proportional to PD as shown in Figure 4 (p≤0.001, r=-0.7).

## Discussion

Our study found that the majority of the participants had a good (187, 55.7%) or very good quality of life (79, 23.5%), with only a small percentage (14, 4.2%) reporting a poor or very poor quality of life. However, significant differences were observed in all quality of life domains between non-dormitory and dormitory students, with non-dormitory students having a higher quality of life score (94.55). Additionally, females were found to have a lower quality of life (90.68) compared to males (96.22). There was a significant correlation between having a roommate and lower QoL scores, but no significant differences were observed based on the city where participants’ families lived.

In terms of psychological distress, the study found that a high percentage of participants were likely to have a psychological disorder (260, 77.6%). This is consistent with the literature that presents high levels of psychological distress among university students (83.9%), higher than those of the general public [14]. Furthermore, compared to our study which found 110 (32.7%) participants predicted to have a severe disorder, previous reports of prevalence rates for depression among medical students have ranged from 15% to 23% [15,16]. This can be explained as university students often face unique stressors related to academic pressure, lifestyle changes, and social demands, which may contribute to elevated levels of psychological distress.

Among our respondents, psychological distress was highest among pharmacy students 39 (43.8%). This goes in accordance with some articles that found the prevalence of psychological distress to be as high as 61.1% among pharmacy students [17]. The College of Medicine is preceded by College of Pharmacy in terms of psychological distress prevalence, followed by the College of Dentistry. This may reflect personal factors such as enhanced coping mechanisms or improved time management or study skills among medical students in particular. Previous research suggests that many medical students feel that their lives are controlled by the medical program and find the curriculum more threatening than challenging [18]. Although most medical students need healthcare, many seek services outside their training institution, consult with peers, or avoid care altogether due to concerns about confidentiality and training schedules [19].

Females were more likely to be in the severe disorder group in this study, this is consistent with the literature that finds the prevalence of conditions like depression and anxiety are higher in female university students (66.6%) compared to male university students (44.4%) [20,21]. Although this might be explained by the disproportionate male-to-female ratio in the sample, which could affect our results. According to the gender distribution of our study sample, 250 participants (74.4%) were female, compared to 86 participants (25.6%) who were male, resulting in a female-to-male ratio of 3:1.

According to this study, visiting one’s family and psychological distress were inversely related. The groups that visited their families more frequently reported overall less severity on the psychological distress scale (28, 19.2%). This can be attributed to the support they might get from interacting with their beloved, as literature does support that familial support is a protective factor against mental illnesses [22]. Furthermore, the literature also states that mental health patients do perceive a lower level of familial support [23].

In our study, individuals who never exercise showed a higher likelihood (135, 40.1%) of belonging to the severe disorder group compared to those engaging in physical activity at least five times a week (84, 25.0%). This finding contrasts with previous research among Chinese adolescents, which highlighted a negative association between regular physical exercise and psychological distress [24]. These results emphasize the need for further investigation into this complex relationship. Another study explains that exercise has a protective function when it comes to psychological distress [25].

This study found an inverse relationship between quality of life and psychological distress with one previous study revealing that if the students are not utilizing effective stress coping skills, they often develop psychological symptoms of distress, resulting in compromised consequences which may end up with lower QoL [18]. This can be understood as the lower the quality of someone’s life is, the more trouble and external negative factors they have to deal with, and thus ultimately, the more distress they are put under.

Limitations

This study carries a few limitations. First, the study is cross-sectional in nature which restricts its ability to establish any causal relationships between psychological distress and quality of life. However, it has been reported that a cross-sectional study may provide insights into the causal relationship between exposure and disease incidence [26]. Secondly, using self-reported data may lead to response bias with participants potentially giving answers that feel more socially acceptable than their actual views. This may affect the validity and accuracy of the findings. Thirdly, the sample size for this study was relatively small, which may limit the generalizability of the results. Given that cross-sectional studies typically benefit from larger sample sizes, it is important to note that smaller samples can be more susceptible to the influence of measurement errors. Another limitation concerning the sample size calculation is that another formula would be more appropriate for comparing two groups (students residing in dormitories vs. those residing at home). Moreover, students with other mental health conditions were not excluded from participation; hence, it may have affected the results of the study. The study was also conducted at a single institution and only on healthcare students, which may not represent the broader population of other majors or even medical students in the UAE or other regions. Therefore, caution should be exercised in interpreting the results.

Recommendations and future implications

Drawing from the research findings, it is imperative to outline a set of recommendations and future implications to address the issues raised in the study. Firstly, universities must place a paramount emphasis on the mental health and well-being of dormitory students. Implementing accessible and comprehensive mental health support services, such as counseling and awareness campaigns, can help mitigate the prevalence of psychological distress. Secondly, promoting healthy lifestyles through fitness programs and sports facilities is essential in reducing psychological distress levels among students, regardless of their living arrangements. Thirdly, fostering social integration among dormitory residents is vital. Universities should facilitate events and clubs that create a sense of community, alleviating the challenges of living away from family and friends. Lastly, a continuous review of dormitory policies, incorporating student feedback, can contribute to a more comfortable living environment. These recommendations point to the importance of proactive measures in safeguarding the well-being of dormitory students. Future research, employing longitudinal and cross-cultural studies, will provide a deeper understanding of the dynamics at play and the effectiveness of interventions. Ultimately, this research illuminates the path toward creating a supportive and conducive learning environment for all students, ensuring their success both academically and personally.

## Conclusions

In conclusion, the results of the study suggest that psychological distress is a prevalent issue among students, with several risk factors identified. This study sheds light on the challenges faced by university students living in dormitories away from their families, highlighting the high prevalence of psychological distress and reduced quality of life. The high prevalence of psychological distress among dormitory students is a cause for concern, with the majority of participants reporting moderate-to-severe psychological distress. This is consistent with previous research that suggests that living in dormitories is associated with higher rates of depression and other mental health issues among university students. It is important for universities to recognize this issue and provide adequate support and resources for their students to help them cope with the challenges of dormitory living. The study also found a significant difference in quality of life between dormitory and non-dormitory students. This is likely the case because dormitory students have to deal with a host of challenges, such as being away from family and friends, adapting to a new lifestyle, and balancing academic and social responsibilities, which can negatively impact their well-being.

This study underscores the need for universities to take proactive measures to ensure that the well-being of dormitory students is not compromised. The study can provide insight into developing targeted interventions and resources to support dormitory students and enhance their overall quality of life. Ultimately, these efforts can lead to a more supportive and conducive learning environment for students contributing to their success as university students and as future healthcare professionals.
